# Interfacial and Bulk Properties of Volatile Amphiphiles and Sodium Dodecyl Sulfate Mixtures

**DOI:** 10.3390/molecules31081256

**Published:** 2026-04-10

**Authors:** Ralitsa Uzunova, Rumyana Stanimirova, Krassimir Danov

**Affiliations:** 1 Department of Chemical & Pharmaceutical Engineering, Faculty of Chemistry & Pharmacy, Sofia University “St. Kliment Ohridski”, 1164 Sofia, Bulgaria; ru@lcpe.uni-sofia.bg (R.U.); rs@lcpe.uni-sofia.bg (R.S.); 2 CoC “Smart Mechatronics, Eco- and Energy Saving Systems and Technologies”, 1164 Sofia, Bulgaria

**Keywords:** benzyl acetate, linalool, geraniol, menthol, citronellol, sodium dodecyl sulfate, surface tension and aqueous solubility of mixtures, bulk and surface pair interaction parameters, physicochemical characterization

## Abstract

Volatile amphiphiles and surfactant mixtures have gained wide applications in diverse areas of industry, cosmetics, and medicine. The surface tension isotherms, measured at different solute ratios, and data processing, using appropriate theoretical models, provide quantitative information on their bulk and interfacial properties. Here, this approach is applied for mixtures of volatile amphiphile (benzyl acetate, linalool, geraniol, menthol, citronellol) and sodium dodecyl sulfate (SDS). All surface tension isotherms are described by the van der Waals model for a two-component adsorption layer, taking into account the counterion binding in the Stern layer, by varying only one adjustable parameter (interfacial pair interaction energy between adsorbed molecules). Knowing the parameters of the model, we computed various properties of the adsorption layers (adsorptions of different components, occupancy of the Stern layer, and interfacial electrostatic potential). The experimental aqueous solubilities of mixtures are fitted using the regular solution theory to obtain the pair bulk interaction parameter. The mixing of SDS and: (i) benzyl acetate and citronellol is antagonistic; (ii) linalool and geraniol is synergistic; and (iii) menthol is ideal. The reported properties of the volatile amphiphiles and SDS mixtures could be of interest for increasing the range of their applicability in practice.

## 1. Introduction

From a chemical viewpoint, fragrances present some of the most complex additives in the formulation of consumer products. Their composition can include more than 100 compounds such as essential oils, their isolates, and various synthetic aroma chemicals [1,2]. Thousands of volatile organic molecules have been investigated by two-dimensional gas chromatography combined with mass spectrometry [3]. Fragrance research has been found to have a major impact on the purchase decision and to be a “primary driver” of branded items in the following categories: body care, skin care, and household care [4]. Volatile compounds are of considerable importance in a variety of industrial, civilian, military, and national security contexts [5,6,7,8,9,10,11,12,13,14,15], as well as in cosmetics [16,17,18,19,20]. Geraniol has been reported to exhibit a variety of pharmacological properties, including anti-inflammatory [21], antimicrobial [22], and antitumor activities [23]. The local application of low concentrations of menthol alleviates pain associated with limb sprains as well as headaches [24] and it is used in the treatment of patients with mild asthma [25].

A common issue for the final products is the fact that the addition of fragrances to the surfactant formulations can significantly influence the physicochemical properties and thus affect the macroscopic appearance even at quite low concentrations. Some fragrance ingredients lead to a change in viscosity, others to clouding phenomena or phase separation [26,27]. Even at relatively low concentrations, fragrance ingredients can markedly modify the properties of foams [28,29,30], liquid detergents [31], and emulsions [32] with consequences for product stability, rheological characteristics, and consumer perception. For example, the addition of 1 wt% linalool, citronellol, or citral (as cosurfactants) to the concentrated 10 wt% mixed solutions of sodium lauryl ether sulphate and cocamidopropyl betaine increases the viscosity of the formulation and respectively decreases the dynamic surface tension of the diluted 0.5 wt% solutions [33,34]. Thus, the mixtures of conventional and volatile amphiphiles suggest that such aroma molecules can be used as cosurfactants to enable interfacial processes and to decrease the amount of the residual surfactants in the resulting products [35].

This work focuses on the characterization of the interfacial and bulk properties of mixed fragrance and sodium dodecyl sulfate (SDS) solutions. The five volatile amphiphilic molecules considered (benzyl acetate, linalool, geraniol, menthol, citronellol) have the following properties: (i) good solubility in alcohols, ethers, and some oils; (ii) low vapor pressures at room temperature of tens of Pa; (iii) low solubility in water; and (iv) the pronounced ability to adsorb at the air/water interface, to reduce the interfacial tension, and alter the interfacial rheology. In the literature [36], the surface tension isotherms of aqueous solutions of 10 monoterpene alcohols are measured using the static method for equilibrium surface tension measurements. The respective surface tension isotherms for benzyl acetate, linalool, geraniol, menthol, and citronellol aqueous solutions measured using the maximum bubble pressure method (MBPM) are published in Refs. [37,38]. Data processing of the isotherms, applying appropriate models for adsorption [36,37,38], reveals information on the excluded area per molecule, adsorption energy, and the effective pair interaction parameter between adsorbed molecules. The concentration, above which the surface tension remains constant, corresponds exactly to the aqueous solubility limit of the respective volatile amphiphile.

The interactions between the fragrance and surfactant molecules can be experimentally characterized using headspace gas chromatographic analysis [39] and neutron reflectivity measurements [40,41,42,43]. The authors [40,41] reported data for the adsorptions of linalool and SDS at the air/solution interface as a function of the mole fractions of linalool in linalool + SDS mixed solutions. They showed that the addition of NaCl suppresses the electrostatic interactions, increases the SDS adsorption and decreases the linalool adsorption. An alternative way to characterize the synergistic interaction between fragrance and surfactant molecules at the air/solution interface is to measure the surface tension isotherms of mixed aqueous solutions at various ratios between solutes [40,44,45,46]. From the experimental surface tension data for linalool + sodium dodecyl 6-benzene sulfonate [40] and linalool + SDS [44] mixed solutions, the authors processed the respective data at a constant surface tension and different solute ratios using the Rosen approach [47] to describe the binary mixed adsorption layers as a regular solution. The Rosen approach has also been applied [46] to characterize the synergistic interactions in the interfacial layer between geraniol and surfactants (anionic SDS, cationic dodecyltrimethylammonium bromide, and nonionic dodecyl ether of poly(23)ethylene glycol). Note that the Rosen theory provides information on the pair interaction between adsorbed species and the composition of the interfacial layer, but not on the magnitudes of the respective adsorptions. To calculate the adsorptions of all species, one needs to simultaneously process all isotherms measured at different concentrations and solute ratios with an appropriate two-dimensional equation of state for the adsorption layer.

One of the most important properties of fragrances is their ability to evaporate from fragrance solutions to the ambient atmosphere and vice versa to condense from fragrance vapors to hydrophilic interfaces. Group-contribution methods have been applied to predict the odor intensity of fragrances [48,49,50]. For mixtures of SDS and linalool, the rate of change in adsorption with time due to forced air flow over a fixed headspace was evaluated using neutron reflectivity over long time scales [43]. An alternative way to characterize the rate of release and evaporation (condensation) of perfume molecules from (to) aqueous solutions is to measure the change in the surface tension vs. time because of the adsorption from vapor (the desorption from fragrance solution) of the volatile amphiphiles [37,38,44,45,51]. From the surface tension isotherms of benzyl acetate, linalool, geraniol, menthol, and citronellol, the instantaneous adsorption and the subsurface concentrations in both phases of the respective volatile amphiphile are calculated. The authors [37,38] have shown that the experimental data for the five volatile amphiphilic molecules are perfectly described by combined mechanisms: convection-enhanced adsorption from water and barrier-controlled desorption from drop to vapor. Therein, the respective adsorption and desorption rate constants are calculated. To apply this approach for mixed fragrance + surfactant solutions, the complete set of the self-consistent physicochemical parameters characterizing the mixed adsorption layers should be obtained.

Our goal in the present study is to characterize the interfacial and bulk properties of the mixed fragrance and SDS aqueous solutions. The combination of the experimental surface tension isotherms, measured for different solute molar ratios (Section 2.2), and their data processing using the generalized van der Waals model for two-component adsorption layers (Section 2.1) provides information on the excluded areas per molecule, the surface pair interaction parameter between all adsorbed species, and the adsorption energies of surfactants, fragrances, and bound counterions. In Section 2.3, the Rubingh theory [52] is applied to describe the dependencies of the critical aggregation concentrations on the molar ratios between fragrances and SDS in order to calculate the bulk interaction parameters for synergistic, ideal, or antagonistic mixing of respective components. The quantitative predictive power of the obtained results and the analysis of the applicability of the Rosen approach [47] for description of the binary mixed adsorption layers as a regular solution are discussed in Section 3. The final conclusions are drawn in Section 5.

## 2. Results

### 2.1. Generalized van der Waals Model for Two-Component Adsorption Layer

Because the used fragrances are partially soluble in water and have pronounced surface activities, the two-component van der Waals model for an ionic–nonionic surfactant mixture [53,54,55] can be used to describe the experimental surface tension isotherms of the anionic surfactant sodium dodecyl sulfate (SDS) in the presence of volatile amphiphiles and NaCl. We will use the following numbering of the species: component 1, a surfactant ion (${DS}^{-}$); component 2, a nonamphiphilic counterion (${Na}^{+}$); component 3, a nonamphiphilic co-ion (${Cl}^{-}$); and component 4, nonionic fragrance molecules. The bulk activities of ionic components, $a_{j}$, are related to the respective concentrations, $c_{j}$, by means of the formula $a_{j}=\gamma_{\pm }c_{j}$ (*j* = 1, 2, 3), with $\gamma_{\pm }$ being the activity coefficient. For the nonionic component, one uses $a_{4}=c_{4}$. The adsorptions of the surfactant ions and the fragrance molecules at the interface are $\Gamma_{1}$ and $\Gamma_{4}$, respectively. The number of the bound counterions per unit area (adsorption of sodium ions) is $\Gamma_{2}$ and the co-ions do not present in the adsorption layer ($\Gamma_{3}=0$). Thus, the occupancy of the Stern layer is simply calculated as $\theta =\Gamma_{2}/\Gamma_{1}$. If *e* is the electronic charge, *k* is the Boltzmann constant, and *T* is the absolute temperature, then it is convenient to work with the dimensionless surface electrostatic potential, $\Phi \equiv e\varphi /\left(kT\right)\geq 0$, where the dimensional surface electrostatic potential is *φ*. It is shown in the literature [56] that 10 mM ${Cl}^{-}$ or ${Br}^{-}$ co-ions added to the SDS solution do not affect the properties of the adsorption layer. In contrast, at a ${Br}^{-}$ concentration of 0.5 M, soluble SDS molecules are strongly expelled to the air–water interface. A strong gauche defect exists among the surfactant hydrophobic tails. An entirely different surfactant packing scheme is observed under the influence of 0.5 M NaCl. The interfacial surfactant molecules suffer a very strong gauche defect among their alkyl chains and are likely to have adopted a more disordered conformation and a horizontal orientation. Thus, our model with $\Gamma_{3}=0$ is adequate for low concentrations of added NaCl, especially for 10 mM NaCl.

The physicochemical parameters of the individually adsorbed ionic surfactant molecules at the surface are: the excluded area of the hard-core interactions between molecules, $\alpha_{11}$; adsorption energy, $E_{1}$; and the effective pair interaction energy between molecules at the surface, $\beta_{11}$. The respective physicochemical parameters of the adsorbed individual fragrance molecules are $\alpha_{44}$, $E_{4}$, and $\beta_{44}$. In the case of mixed adsorption layers, the total adsorption is $\Gamma =\Gamma_{1}+\Gamma_{4}$. The parameters $\beta_{14}$ and $\alpha_{14}$ are respectively the effective pair interaction energy and the excluded area of the hard-core interactions between surfactant and fragrance molecules at the surface. The effective pair interaction energies clump together all multiple contributions (hydrophobic interactions, chain conformation effects, etc.) up to the second virial expansion of the total surface free energy [54]. In the framework of the van der Waals model, $\alpha_{14}$ and the average excluded area, *α*, are defined as follows: $4\alpha_{14}\equiv \alpha_{11}+2{\left(\alpha_{11}\alpha_{44}\right)}^{1/2}+\alpha_{44}$ and $\Gamma^{2}\alpha \equiv \Gamma_{1}^{2}\alpha_{11}+2\Gamma_{1}\Gamma_{4}\alpha_{14}+\Gamma_{4}^{2}\alpha_{44}$. The positive values of the effective pair interaction energy correspond to attraction and the negative values to repulsion between adsorbed molecules.

At equilibrium, the chemical potentials in the bulk and at the surface of the respective components (surfactant ions and fragrance molecules) are equal, so that the expressions for the adsorption isotherms read:

(1) $$\exp\left(\frac{E_{j}}{kT}\right)\upsilon_{j}a_{j}=f_{j}\alpha_{jj}\Gamma_{j}\;(j=1,\;4),$$

where $f_{j}$ is the surface activity coefficient and $v_{j}$ is the molecular volume of the *j*-th component. The surface activity coefficients, $f_{j}$, account for the interactions between molecules at the surfaces (hard-core, electrostatic, and effective pair interaction energy) and they are calculated from the following expressions [54]:

(2) $$f_{1}=\frac{1-\theta }{1-\Gamma \alpha }\exp\left[\frac{\Gamma_{1}\left(2\alpha_{11}-\alpha \right)+\Gamma_{4}\left(2\alpha_{14}-\alpha \right)}{1-\Gamma \alpha }-\frac{2}{kT}\left(\Gamma_{1}\beta_{11}+\Gamma_{4}\beta_{14}\right)+\Phi \right],$$

(3) $$f_{4}=\frac{1}{1-\Gamma \alpha }\exp\left[\frac{\Gamma_{1}\left(2\alpha_{14}-\alpha \right)+\Gamma_{4}\left(2\alpha_{44}-\alpha \right)}{1-\Gamma \alpha }-\frac{2}{kT}\left(\Gamma_{1}\beta_{14}+\Gamma_{4}\beta_{44}\right)\right].$$

The counterions bind to the ionic surfactant headgroups, so that the localized Langmuir type adsorption isotherm, written for the occupancy of the Stern layer *θ*, yields:

(4) $$\exp\left(\frac{E_{2}}{kT}+\Phi \right)v_{2}a_{2}=\frac{\theta }{1-\theta },$$

where $v_{2}$ is the volume of the hydrated sodium ion. The surface charge-balance equation (the well-known Gouy equation),

(5) $$\Gamma_{1}-\Gamma_{2}={\left(\frac{2c_{2}}{\pi \lambda }\right)}^{1/2}\sinh\left(\frac{\Phi }{2}\right),$$

closes the system of equations, Equations (1)–(4). Here, *λ* = 0.715 nm is the Bjerrum length. For given physicochemical parameters and bulk concentrations, the model predicts: the adsorptions $\Gamma_{1}$, $\Gamma_{2}$, and $\Gamma_{4}$; occupancy of the Stern layer, *θ*; and the surface electrostatic potential, *φ*. The activity coefficient, $\gamma_{\pm }$, and the Bjerrum length are calculated from Equations (S1) and (S2). Finally, the equilibrium surface tension, *σ*, is calculated from the two-dimensional equation of state [53,54,55]:

(6) $$\sigma =\sigma_{0}-kT\frac{\Gamma }{1-\Gamma \alpha }+\beta_{11}\Gamma_{1}^{2}+2\beta_{14}\Gamma_{1}\Gamma_{4}+\beta_{44}\Gamma_{4}^{2}-2kT\left(\Gamma_{1}-\Gamma_{2}\right)\tanh\left(\frac{\Phi }{4}\right),$$

where $\sigma_{0}$ is the surface tension of the pure solvent. The last term in the right-hand side of Equation (6) represents the contribution of the diffuse part of the electric double layer to the surface tension [53].

The limiting case of $c_{1}=c_{2}=c_{3}=0$ leads to the adsorption model for single fragrance solutions used to process experimental data for the surface tension isotherms of studied volatile amphiphiles [37,38]. The opposite case of $c_{4}=0$ is used to process the surface tension isotherms of SDS in the presence of NaCl [53,54].

### 2.2. Adsorptions from Mixed SDS–Fragrance Solutions

It is well-known in the literature [54,57], that the presence of small amount of dodecanol in the samples of SDS considerably affects the values of the equilibrium surface tension measured by the maximum bubble pressure method (MBPM) and by the static methods using the du Noüy ring and the Wilhelmy plate methods. The presence of NaCl suppresses the effect of dodecanol admixture.

**Figure 1:**
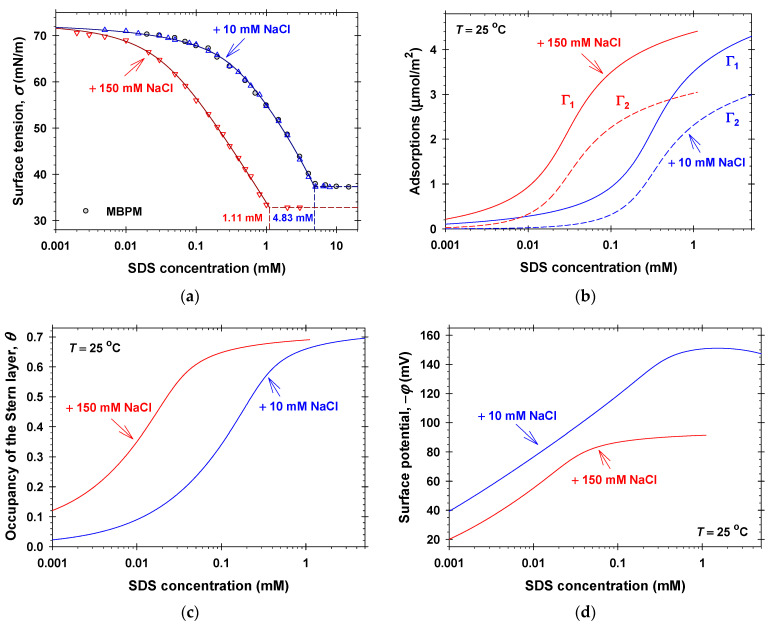
(**a**) Surface tension isotherms of SDS solutions in the presence of 10 mM and 150 mM NaCl: empty symbols correspond to *σ* measured using the static methods [58]; solid symbols show experimental values of *σ* obtained from the MBPM; solid lines show the calculated best fit results. Calculated (**b**) surfactant and counterion adsorptions, (**c**) occupancy of the Stern layer, and (**d**) surface electrostatic potentials vs. SDS concentration in the presence of 10 mM and 150 mM NaCl.

To check the correctness of the surface tension isotherms obtained by the MBPM, we measured *σ* vs. the SDS concentration in the presence of 10 mM NaCl and calculated the equilibrium surface tension by applying the long-time asymptotic approach (see Section 4). Figure 1a shows the isotherms of SDS solutions in the presence of 10 mM and 150 mM NaCl. The empty symbols therein correspond to *σ* measured using the static methods [58] and the solid symbols show experimental values of *σ* obtained by the MBPM. The surface tension isotherms of aqueous solutions of linalool, citronellol, geraniol, and menthol are measured using the static method [36]. The respective surface tension isotherms of linalool and citronellol [37] and of geraniol and menthol [38] aqueous solutions, applying the long-time asymptotic approach, coincide with those measured using the static method. The coincidence of the experimental data for SDS + 10 mM NaCl and for fragrance aqueous solutions from both methods suggests that the used MBPM approach is correct and can be successfully applied to measure the equilibrium surface tensions of mixed SDS + fragrance solutions in the presence of 10 mM NaCl.

The solid lines in Figure 1a show the calculated surface tension vs. concentration from the best fit of all experimental data (symbols in Figure 1a) using the van der Waals model for ionic surfactants (Section 2.1). The obtained physicochemical parameters are: $E_{1}$ = 12.6 ± 0.1 *kT*; $E_{2}$ = 1.74 ± 0.02 *kT*; $\alpha_{11}$ = 0.30 ± 0.01 nm^2^; and $\beta_{11}$ = 2.10 ± 0.05 $\alpha_{11}$*kT*. The regression coefficient was 0.9998. The values of the critical micelle concentrations, $C_{1}$, are 4.83 mM in the presence of 10 mM NaCl and 1.11 mM in the presence of 150 mM NaCl. The calculated dependencies of the surfactant and counterion adsorptions, the occupancy of the Stern layer, and the surface electrostatic potential on the SDS and NaCl concentrations are summarized in Figure 1b and Figure 1c, and Figure 1d, respectively. The increase in the SDS concentration leads to the increase in both surfactant and counterion adsorptions, $\Gamma_{1}$ and $\Gamma_{2}$, the occupancy of the Stern layer, the magnitude of the surface electrostatic potential, and the number of surface charges per unit area (surface charge density). The increase in NaCl concentration decreases the magnitude of the surface potential and as a result, it leads to the pronounced increase in counterion binding. Note that the increase in the occupancy of the Stern layer is due to the increase in both $\Gamma_{1}$ and $\Gamma_{2}$ and, as a result, the surface charge density increases.

**Figure 2:**
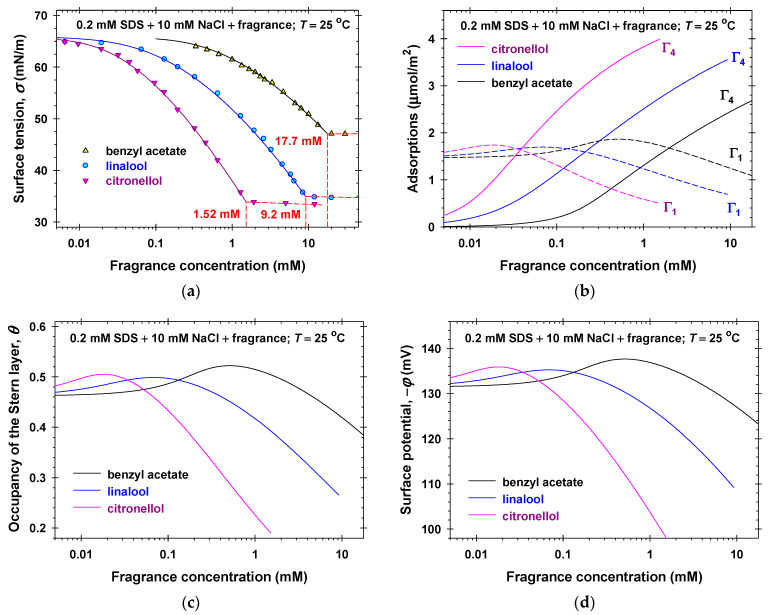
(**a**) Surface tension isotherms of 0.2 mM SDS + 10 mM NaCl and fragrance (benzyl acetate, linalool, and citronellol) mixed solutions: symbols correspond to experimental data; solid lines show the calculated best fit results. Calculated (**b**) surfactant and fragrance adsorptions, (**c**) occupancy of the Stern layer, and (**d**) surface electrostatic potentials vs. fragrance concentrations.

**Figure 3:**
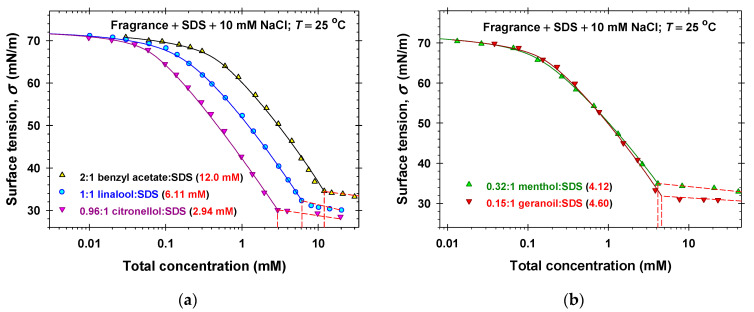
Surface tension isotherms of fragrance + SDS + 10 mM NaCl mixed solutions at fixed molar ratios between fragrance and SDS: (**a**) benzyl acetate, linalool, and citronellol; (**b**) menthol and geraniol. The symbols denote experimental data and the solid lines represent the best fits obtained using our model.

**Figure 4:**
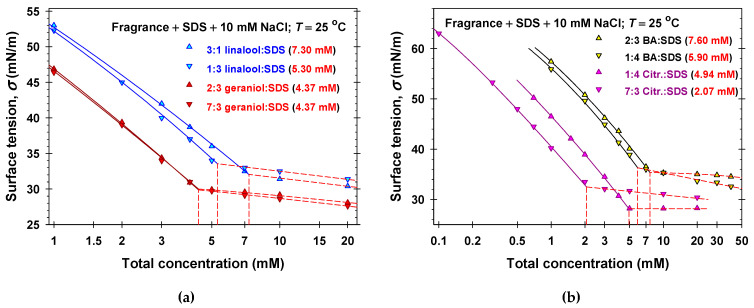
Surface tension isotherms of fragrance + SDS + 10 mM NaCl mixed solutions at fixed molar ratios between fragrance and SDS: (**a**) linalool and geraniol; (**b**) benzyl acetate (BA) and citronellol (Citr.). The symbols denote experimental data and the solid lines represent the best fits obtained using our model.

To characterize the interfacial and bulk properties of the mixed fragrance + SDS solutions, we applied the following strategy. First, we measured the equilibrium surface tensions of 0.2 SDS mM + 10 mM NaCl solutions in the presence of different amounts of volatile amphiphilic components (Figure 2a and Figure S5a). Second, the complete surface tension isotherms of the mixture between fragrances and SDS at fixed molar ratios between components were determined (Figure 3). Finally, we varied the molar ratios between fragrance and SDS and measured *σ* for a set of total solute concentrations, $c=c_{1}+c_{4}$, to obtain the respective critical aggregation concentrations, *C* (Figure 4 and Figure S7). For total solute concentrations, *c* > *C*, the values of the experimental surface tension are approximately constant, which shows that the bulk chemical potentials of monomers remain constant.

The interfacial physicochemical parameters of individual fragrances (excluded area, $\alpha_{44}$, adsorption energy, $E_{4}$, and effective pair interaction energy, $\beta_{44}$, of adsorbed molecules) are known, as seen in Table 1 and Refs. [37,38]. The respective parameters for SDS are determined above (Figure 1). Thus, the only unknown value in our model is that for the effective pair interaction energy between surfactant and fragrance molecules at the surface, $\beta_{14}$.

We simultaneously fitted all experimental isotherms (symbols in Figure 2a, Figure 3, Figure 4, Figures S5a and S7) for a given volatile amphiphilic component and SDS at concentrations *c* < *C* by varying only one adjustable parameter, $\beta_{14}$. In all cases the values of the regression coefficients were larger than 0.9995 and the agreement between the experiment and the theory was excellent. The obtained best fit values of $\beta_{14}$ are listed in Table 1 and the calculated theoretical dependencies of *σ* vs. concentrations are plotted in Figure 2a, Figure 3, Figure 4, Figures S5a and S7 (solid lines).

Note that for non-cyclic aromatic/terpene molecules, the values of the effective interaction energies, $\beta_{14}$, increase in the order: linalool < citronellol < geraniol. The strongest interaction is observed between SDS and geraniol, due to its two double bonds and a hydroxyl (OH) group attached to a primary carbon atom at the beginning of the chain. In comparison, citronellol, which has only one double bond, shows lower $\beta_{14}$ values. Linalool exhibits the lowest values, because its OH group is attached to a tertiary carbon atom, leading to greater steric hindrance and less efficient packing in the adsorption layers [59,60]. In the case of cyclic molecules, a stronger interaction is observed between SDS and benzyl acetate than between SDS and menthol. This difference is mainly determined by their molecular structure. Benzyl acetate has a planar aromatic ring, which allows it to align easily between the hydrocarbon tails of SDS, whereas menthol contains a bulky, three-dimensional cyclohexane ring that introduces significant steric hindrance. In addition, benzyl acetate is less branched, while menthol possesses bulky substituents that further hinder tight packing. Benzyl acetate can participate in π-interactions and exhibits better polar adaptability, whereas menthol relies primarily on weaker van der Waals forces [61,62,63]. Overall, benzyl acetate is incorporated more efficiently and interacts more strongly with SDS due to its flatter and less bulky structure, while menthol is more sterically hindered and packs less effectively.

For all studied fragrances, the values of the effective pair interaction energy between adsorbed volatile amphiphilic molecules and SDS molecules, $\beta_{14}$, are larger than those of individual molecules $\beta_{11}$ and $\beta_{44}$. Thus, the effective attractions between adsorbed SDS and fragrance molecules are stronger than those between the respective individual adsorbed molecules. The considerable attraction interactions between linalool and sodium dodecyl 6-benzene sulfonate [40] and between linalool and SDS [41] molecules in the mixed adsorption layers were also measured by neutron reflectivity.

For the precise calculation of the critical aggregation concentration (CAC) of the mixed solutions, *C*, we used the intersection point of the calculated theoretical dependence of *σ* vs. the total concentration *c* for *c* < *C* (solid lines) with the interpolation curves, *σ* vs. *c*, for *c* > *C*. The obtained values of the CAC are listed in Figure 2a and Figure S5a and in the legends of Figure 3 and Figure 4, and Figure S7. The experimental dependencies of *C* vs. molar fractions of SDS are used as a basic data set for the application of the regular solution theory in Section 2.3.

Having determined the physicochemical parameters of the generalized van der Waals model once (Section 2.1), we are able to calculate various properties of the mixed adsorption layers, such as: the surfactant adsorption, $\Gamma_{1}$, the bound counterion adsorption, $\Gamma_{2}$, and the fragrance adsorption, $\Gamma_{4}$; the occupancy of the Stern layer by adsorbed counterions, *θ*; the surface electrostatic potential, *φ*; etc. Figure 2b–d and Figure S5b–d summarize the respective calculated data for 0.2 mM SDS + 10 mM NaCl and fragrance mixed solutions at *c* < *C*. As should be, the adsorption of SDS, $\Gamma_{1}$, is higher than that of the volatile amphiphilic molecules, $\Gamma_{4}$, at low fragrance concentrations and it becomes lower, $\Gamma_{1}<\Gamma_{4}$, with the rise in the fragrance concentration. The concentrations, $c_{14}$, at which $\Gamma_{1}=\Gamma_{4}$, are ordered as follows: 1.95 mM for benzyl acetate; 0.208 mM for linalool; 0.120 mM for menthol; 0.111 mM for geraniol; and 0.037 mM for citronellol. This order correlates to the adsorption energies of fragrances (Table 1)—the higher the adsorption energies the lower the fragrance concentrations are. Note that linalool and geraniol are isomers with equal energies of adsorptions. Nevertheless, the higher attraction interaction energies, $\beta_{14}$ and $\beta_{44}$, of geraniol compared to those of linalool (Table 1) lead to a two times lower concentration, $c_{14}$, for geraniol. The displacement of the adsorbed anionic surfactant molecules with the adsorbed linalool molecules was also observed experimentally in Refs. [40,41].

Because of the fixed ionic strength (10.2 mM), the magnitude of the surface electrostatic potential excellently correlates with the occupancy of the Stern layer, as seen in Equation (5) and Figure 2c,d and Figure S5c,d. The partial displacement of the adsorbed SDS molecules for $c_{14}<c_{4}$ leads to the decrease in the magnitudes of the surface charge density and the surface electrostatic potential. The change in *φ* is the highest (more than 40 mV) in the case of citronellol and the lowest (only 14 mV) in the case of benzyl acetate adsorption.

**Figure 5:**
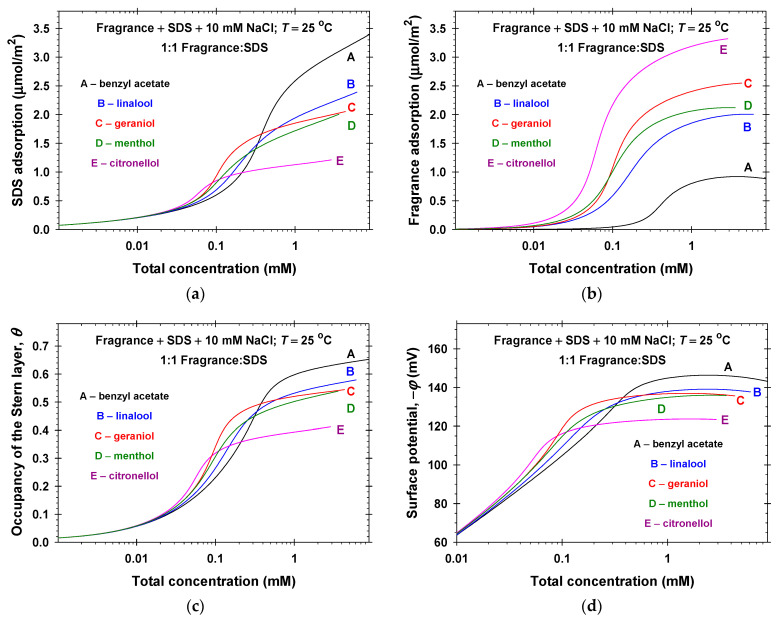
Calculated dependencies of (**a**) the surfactant adsorption, (**b**) the fragrance adsorption, (**c**) the occupancy of the Stern layer, and (**d**) the surface electrostatic potentials on the total concentration for fragrance + SDS + 10 mM NaCl solutions at a 1:1 molar ratio between the fragrance and SDS.

Figures S6 and S8 summarize the calculated properties of the mixed adsorption layers in the case of the fixed molar ratios of SDS and fragrance solutions reported in Figure 3. Because of the used different ratios of the respective fragrance and SDS, which were convenient for the measurements of the surface tension isotherms, the conclusions for the general trends are difficult to clearly draw. For that reason, we calculated $\Gamma_{1}$, $\Gamma_{4}$, *θ*, and *φ* for fragrance + SDS + 10 mM NaCl solutions at the equimolar molar ratio between the solutes (Figure 5). It is obvious, that the saturated fragrance adsorptions (3.32 $\mu mol/m^{2}$ for citronellol, 2.55 $\mu mol/m^{2}$ for geraniol, 2.12 $\mu mol/m^{2}$ for menthol, 2.01 $\mu mol/m^{2}$ for linalool, and 0.88 $\mu mol/m^{2}$ for benzyl acetate) correlate well with the adsorption energies, $E_{4}$—the larger the adsorption energy the higher the volatile amphiphile adsorptions are (see Figure 5b and Table 1). Note that the adsorption energy of menthol is about 0.5 *kT* higher than that of geraniol, but because menthol has a larger excluded area per molecule and lower effective interactions energies, $\beta_{14}$ and $\beta_{44}$, the menthol saturation adsorption becomes lower than that of geraniol (Figure 5b). The saturation adsorptions of the anionic surfactant in the presence of the respective fragrance are oppositely ordered (Figure 5a). Note that the anionic surfactant adsorption at total concentration *C* is higher for benzyl acetate, linalool, and geraniol and is lower for menthol and citronellol than the respective fragrance adsorption. The calculated values of the SDS and linalool adsorptions are close to the respective experimental data measured by neutron reflectivity, as seen in [41] and Section 3.

As can be expected, the occupancy of the Stern layer and the magnitude of the surface electrostatic potential increase with the increase in the total concentration of solutes because of the increase in the SDS adsorption (Figure 5c,d). The effect of the fragrance type on the occupancy of the Stern layer at total species concentration *C* is more pronounced—*θ* drops by more than three times when changing benzyl acetate with citronellol. Nevertheless, the respective surface electrostatic potential of the equimolar mixed solution of SDS + benzyl acetate of −143 mV increases only to −123 mV in the case of equimolar mixed solutions of SDS + citronellol.

From the experimental values of the SDS critical micelle concentration, $C_{1}$, the fragrance solubility in water, $C_{4}$, and the critical aggregation concentration of mixed SDS–fragrance solutions, *C*, one can obtain the SDS–fragrance bulk interaction parameter, *β*, appearing in the regular solution theory.

### 2.3. Bulk Properties of Mixed SDS–Fragrance Solutions

The regular solution theory applied to the aggregation in binary mixtures is known as the Rubingh theory [52,64,65]. In fact, the phase separation model is focused on the equilibrium between aggregates and monomers with respect to the exchange of each component in a binary mixture. One uses the following notations: the mole fraction of SDS in the aggregate pseudo-phase is $y_{1}$ and the activity coefficient therein is $\gamma_{1}$; those of the volatile amphiphilic molecules are $y_{4}$ and $\gamma_{4}$, respectively; and $y_{1}+y_{4}=1$. The chemical equilibrium between monomers and aggregate pseudo-phase with respect to the exchange of molecules types “1” and “4” yields [52,64,65]:

(7) $$\ln\left(x_{1}C\right)=\ln C_{1}+\ln\left(\gamma_{1}y_{1}\right);\;\ln\left(x_{4}C\right)=\ln C_{4}+\ln\left(\gamma_{4}y_{4}\right).$$

In the Rubingh theory, only the pair interactions between molecules in the aggregate pseudo-phase are accounted for—the second virial expansion of the free energy is postulated. Thus, the activity coefficients are expressed in the form [52,65,66]:

(8) $$\gamma_{1}=\exp\left(\beta y_{2}^{2}\right);\;\gamma_{2}=\exp\left(\beta y_{1}^{2}\right).$$

In Equation (8), the interaction parameter, *β*, is defined as follows:

(9) $$\beta \equiv \frac{n}{2kT}\left(2w_{14}-w_{11}-w_{44}\right).$$

Here: $w_{11}$ and $w_{44}$ are the energies of interactions of the two closest anionic surfactant and fragrance molecules, respectively; $w_{14}$ is the energy of interactions between the two closest anionic surfactant and fragrance molecules; and *n* is the average number of closest neighbors.

As a general rule, the interaction energies of the two closest neighbors ($w_{11}$, $w_{14}$, and $w_{44}$) are negative (attraction), as seen in Section 2.2 and Table 1. However, *β* can be either negative, positive, or zero. If *β* = 0, then the aggregates represent an ideal mixture of the constituent components. For *β* < 0 (or *β* > 0), we are dealing with negative (positive) deviations from Raoult’s law, i.e., with synergism (antagonism) of the anionic surfactant and fragrance in the mixed aggregates. Note that *β* is a physicochemical parameter which does not depend on the concentration and composition of the binary mixtures.

**Figure 6:**
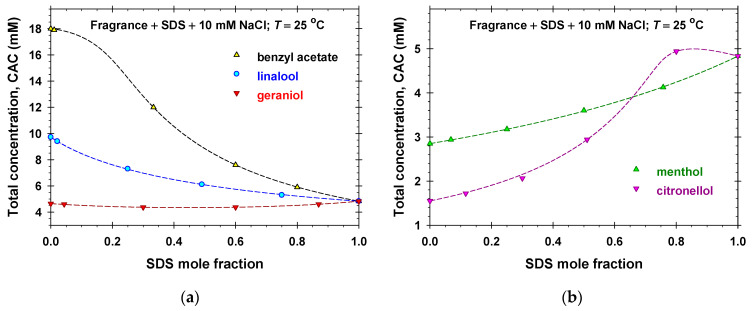
Dependence of the critical aggregation concentration (CAC) of fragrance + SDS + 10 mM NaCl mixed solutions on the mole fraction of SDS: (**a**) benzyl acetate, linalool, and geraniol; (**b**) menthol and citronellol. The symbols are experimental data and the dashed lines represent the best fits obtained using the regular solution theory.

Figure 6 shows the experimental dependencies of the critical aggregation concentrations measured for mixed fragrance + SDS + 10 mM solutions on the mole fraction of SDS. The values of respective concentrations for individual components, $C_{1}$ and $C_{4}$, are known. Thus, the only unknown parameter in the system of equations, Equations (7) and (8), is the bulk interaction parameter, *β*. The dashed lines in Figure 6 correspond to the best fit result obtained using the respective values of *β* listed in Table 1. It is seen that the Rubingh theory excellently describes the experimental data with the values of the regression coefficients greater than 0.9997 and constant interaction parameter *β* for each separate volatile amphiphile. It is interesting to note that the strongest antagonistic mixing is observed for citronellol + SDS (*β* = 1.60), followed by benzyl acetate + SDS (*β* = 1.17) mixed solutions. The mixing of menthol and SDS + 10 mM NaCl is ideal and the respective Raoult’s law takes place. For the isomers (linalool and geraniol), the synergistic mixing with anionic surfactant in the presence of 10 mM NaCl is obtained, *β* < 0 (see Table 1).

**Figure 7:**
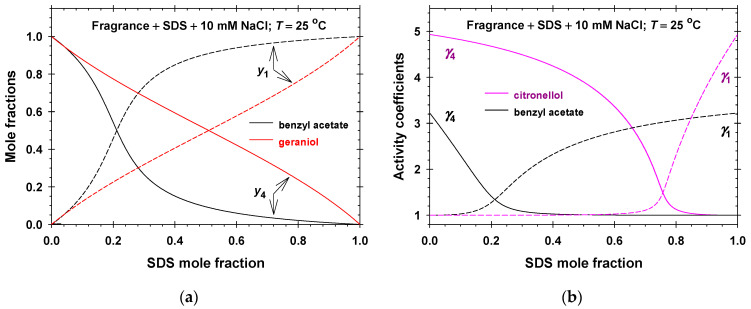
(**a**) Mole fractions, $y_{1}$ and $y_{4}$, vs. SDS bulk mole fractions for benzyl acetate + SDS and geraniol + SDS mixed solutions. (**b**) Activity coefficients, $\gamma_{1}$ and $\gamma_{4}$, vs. SDS bulk mole fractions for menthol + SDS and citronellol + SDS mixed solutions.

The calculated values of the mole fractions of the anionic surfactant, $y_{1}$, and the fragrance molecules, $y_{4}$, in the aggregate pseudo-phase as functions of the SDS bulk mole fraction, $x_{1}$, are plotted in Figure 7a and Figure S9a. Note that the pseudo-phases have equimolar compositions of molecules at increasing SDS bulk mole fractions in the order benzyl acetate (the lowest, $x_{1}$ = 0.211), linalool ($x_{1}$ = 0.332), geraniol ($x_{1}$ = 0.510), menthol ($x_{1}$ = 0.629), and citronellol (the highest, $x_{1}$ = 0.758). The respective dependencies of the activity coefficients, $\gamma_{1}$ and $\gamma_{4}$, of components in the aggregate pseudo-phase vs. SDS bulk mole fractions are illustrated in Figure 7b and Figure S9b. As should be: $\gamma_{1}\geq 1$ and $\gamma_{4}\geq 1$ in the case of antagonistic mixing of citronellol (benzyl acetate) and SDS with a maximum value of 4.93 for citronellol + SDS + 10 mM NaCl; $\gamma_{1}=\gamma_{4}=1$ because of the ideal mixing of menthol and SDS; and $\gamma_{1}\leq 1$ and $\gamma_{4}\leq 1$ in the case of synergistic mixing of geraniol (linalool) and SDS with a minimum value of 0.707 for geraniol + SDS in the presence of 10 mM NaCl.

## 3. Discussion

**Figure 8:**
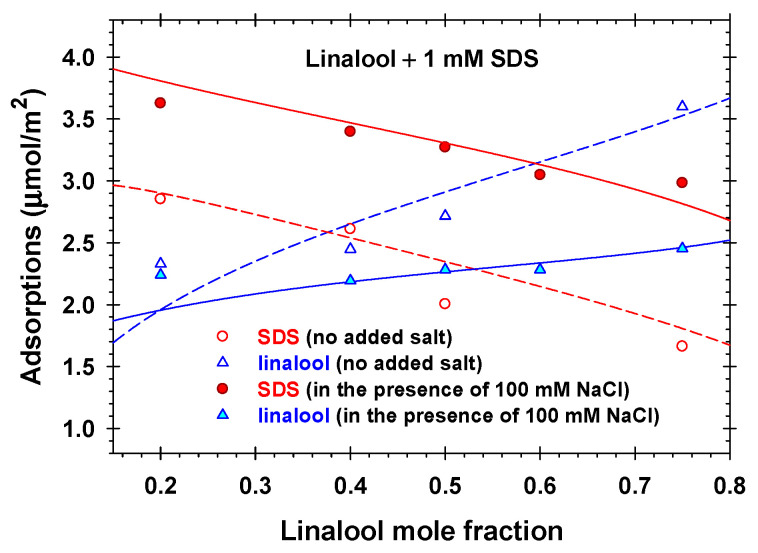
Experimental dependencies (symbols) of the anionic surfactant, $\Gamma_{1}$, and linalool, $\Gamma_{4}$, adsorptions on the mole fractions of linalool measured for linalool + 1 mM SDS solutions in the presence of 100 mM NaCl and without added salt [41]. Adsorptions calculated by means of our model using the parameters listed in Table 1: solid lines—in the presence of 100 mM NaCl; dashed lines—without added electrolyte.

The obtained interfacial physicochemical parameters for the studied volatile amphiphilic molecules (Table 1) and SDS enable the possibility to model the compositions of the adsorption layers and the surface electrostatic potentials for all ratios between solutes in the presence of NaCl or without added salt. To our knowledge, the only experimental data for the adsorptions of linalool and SDS at the air/solution interfaces measured directly by neutron reflectivity are those given in Ref. [41]. The authors reported data for $\Gamma_{1}$ and $\Gamma_{4}$ as functions of the mole fraction of linalool in the linalool + 1 mM SDS mixed solutions in the presence of 100 mM NaCl and without added salt (Figure 8). Using the already determined interfacial physicochemical parameters for linalool and SDS (Table 1), we calculated the adsorptions of linalool and anionic surfactant without any adjustable parameters. The calculated results are plotted in Figure 8. The solid lines therein correspond to the case of added 100 mM NaCl, whereas the dashed lines correspond to the case without added electrolyte. One sees that the agreement between the theoretical predictions and the experimental data for 100 mM added NaCl is excellent. The addition of 100 mM NaCl suppresses the electrostatic interactions, resulting in increased anionic surfactant adsorption and decreased linalool adsorption compared to the case without added salt. Even at a three-time larger linalool concentration (3 mM) compared to the SDS concentration (1 mM), linalool adsorption is lower than the anionic surfactant adsorption. The deviations of the experimental points from the theoretical curves in the case without added NaCl are more pronounced. Nevertheless, the theoretical predictions qualitatively explain these experimental data. It is obvious that the increase in the linalool concentration leads to the partial displacement of the SDS molecules from the adsorption layer and to the higher adsorption of linalool. The good agreement between the theoretical calculations and the experimental results shows that the proposed theoretical model with the reported physicochemical parameters for the fragrance + SDS mixtures have a predictive power.

**Figure 9:**
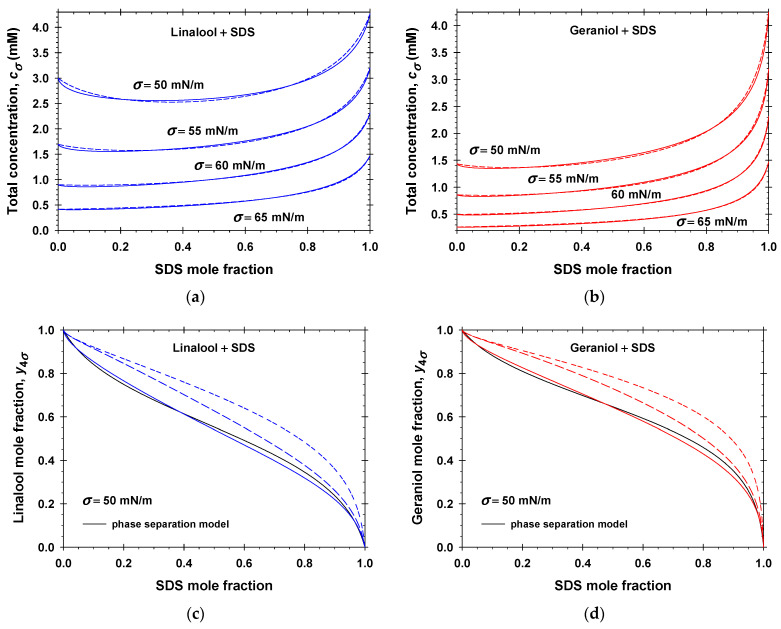
Comparison between the calculated concentrations, $c_{\sigma }$, using our model (solid lines) and those predicted by the best fits using the phase separation model (dashed lines) for mixed adsorption layers of (**a**) linalool + SDS solutions and (**b**) geraniol +SDS solutions. Fragrance mole fraction in the adsorption layers, $y_{4\sigma }$, vs. the SDS bulk mole fraction, $x_{1}$, for (**c**) linalool + SDS and (**d**) geraniol +SDS. Different curves correspond to different definitions of $y_{4\sigma }$ (for the explanation of the meaning of different curves see the main text).

The Rubingh phase separation model [52] was also applied to binary mixed adsorption layers [47] in the case of a constant two-dimensional pressure (i.e., constant surface tension *σ*). We called this approach “the phase separation model for adsorption layers” (PSMAL). Following the PSMAL, the total concentration of solutes at which *σ* is a constant, $c_{\sigma }$, becomes a function of the bulk mole fraction of SDS, $x_{1}$. All kinds of interactions between fragrance and anionic surfactant molecules in the adsorption layers are characterized by the surface interaction parameters, $\beta_{\sigma }$. The experimental values of $\beta_{\sigma }$ are reported in the literature for linalool + SDS [44] and geraniol + SDS [46]. To illustrate the applicability of the PSMAL, we calculated the dependencies of $c_{\sigma }$ on $x_{1}$ using our model for linalool + SDS (solid lines in Figure 9a) and geraniol + SDS (solid lines in Figure 9b) aqueous solutions without added NaCl at different constant values of the surface tension: 50 mN/m; 55 mN/m; 60 mN/m; and 65 mN/m. Subsequently, these calculated data were fitted using the PSMAL (see the dashed lines in Figure 9a,b, Equations (S3) and (S4)). It is seen that the comparison between the two models is quite good. The obtained best fit values of the respective surface interaction parameters, $\beta_{\sigma }$, for linalool + SDS are as follows: −1.29 ± 0.01 at *σ* = 50 mN/m; −1.24 ± 0.01 at *σ* = 55 mN/m; −1.16 ± 0.01 at *σ* = 60 mN/m; and −1.03 ± 0.01 at *σ* = 65 mN/m. In the case of mixed geraniol + SDS adsorption layers, we obtained: −1.66 ± 0.01 at *σ* = 50 mN/m; −1.59 ± 0.01 at *σ* = 55 mN/m; −1.46 ± 0.01 at *σ* = 60 mN/m; and −1.18 ± 0.01 at *σ* = 65 mN/m. The best fit values of $\beta_{\sigma }$ for both fragrances are close to the experimental data reported in Refs. [44,46]. The negative values of the surface interaction parameters correspond to synergistic mixing of fragrances and anionic surfactant molecules in the adsorption layers. Note that the calculated values of $\beta_{\sigma }$ from the definition given by Equation (S5) and the respective parameters listed in Table 1 are −1.23 for linalool and −1.59 for geraniol. These values are close to the best fit parameters obtained for the surface interaction parameters. Thus, the simpler phase separation model can be used to estimate the interaction between adsorbed molecules and the total concentrations, $c_{\sigma }$, by processing the respective experimental data. Nevertheless, collecting enough data for $c_{\sigma }\;vs\;x_{1}$ at a constant value of the surface tension requires considerable amount of experimental effort.

The composition of the adsorption layer in the PSMAL is characterized by the fragrance mole fraction, $y_{4\sigma }$. The black solid lines in Figure 9c and Figure S10 correspond to the calculated values of $y_{4\sigma }$ in the case of linalool + SDS, and those in Figure 9d and Figure S11 are for the geraniol + SDS adsorption layers. In the PSMAL, the binary mixed adsorption layers are composed of fragrance molecules (component 4) and all other adsorbed species are considered as component 1. To clarify the meaning of component 1, we used our model and calculated $y_{4\sigma }$ as $\Gamma_{4}/\left(\Gamma_{1}+\Gamma_{4}\right)$; that is, the adsorption layer in the PSMAL contains only the adsorbed molecules of the anionic surfactant and fragrance (short-dashed lines in Figure 9c,d, Figures S10 and S11). In all studied cases, the short-dashed lines deviate considerably from the predictions of the PSMAL. If one assumes that the bound counterions are also included in the adsorption layer, then $y_{4\sigma }$ should be $\Gamma_{4}/\left(\Gamma_{1}+\Gamma_{2}+\Gamma_{4}\right)$, the long-dashed lines in Figure 9c,d, Figures S10 and S11. One sees that the PSMAL predictions become closer to the respective long-dashed lines, but again $y_{4\sigma }$ is systematically lower. Finally, if one includes both the diffuse electric double layer and adsorption layer in the interfacial pseudo-phase, then the total number of the fragrance molecules per unit area is approximately equal to $\Gamma_{4}$ and the sum of all ionic compounds (${DS}^{-}$, ${Na}^{+}$, and ${Cl}^{-}$) per unit area is approximately equal to ${2\Gamma }_{1}$ because of the electroneutrality. Thus, $y_{4\sigma }$ should be approximately equal to $\Gamma_{4}/\left({2\Gamma }_{1}+\Gamma_{4}\right)$—the colored solid lines in Figure 9c,d, Figures S10 and S11. These curves are very close to the predictions of the PSMAL. Therefore, the interfacial phase considered in the PSMAL contains the adsorption layer and the contiguous electrostatic double layer.

The following general conclusions can be drawn. The PSMAL predicts the synergistic interactions between the anionic surfactant and fragrance molecules and the composition of the interfacial phase relatively well. The main disadvantage is that the PSMAL does not provide information on the magnitude of the fragrance adsorption. To calculate all the properties of the mixed adsorption layer (the adsorptions $\Gamma_{1}$, $\Gamma_{2}$, and $\Gamma_{4}$; occupancy of the Stern layer, *θ*; and the surface electrostatic potential, *φ*), one needs to apply the general model described in Section 2.1.

## 4. Materials and Methods

We studied a wide range of volatile amphiphilic molecules (benzyl acetate, linalool, geraniol, menthol, citronellol) which possess low saturated vapor pressures, appreciable solubilities in water, and well pronounced surface activities. Aqueous mixtures of a given fragrance and anionic surfactant SDS of different ratios were used. The chemical structures of the molecules are shown in Figure S1. All measurements were performed at 25 °C and the fragrance–surfactant aqueous mixtures were prepared with deionized water purified by the Elix 3 water purification system (Millipore, Burlington, MA, USA). The specific resistivity of deionized water was 18.2 MΩ·cm.

The benzyl acetate was a product of Sigma Aldrich, Merck KGaA, Burlington, MA, USA (>99%, Cat. №. A0022) with molecular weight $M_{w}$ = 150.18 g/mol; density *ρ* = 1054 g/dm^3^; specific volume ${1/v}_{4}$ = 7.02 M; and solubility limit in water $C_{4}$ = 18 mM. The linalool was a product of Sigma Aldrich, Merck KGaA, Burlington, MA, USA (>97%, Cat. №. L2602): $M_{w}$ = 154.25 g/mol; *ρ* = 863 g/dm^3^; ${1/v}_{4}$ = 5.59 M; and $C_{4}$ = 9.72 mM. The geraniol was a product of Sigma Aldrich, Merck KGaA, Burlington, MA, USA (>98%, Cat № 163333) with the same molecular weight as that of linalool, $M_{w}$ = 154.25 g/mol, but a different structure (see Figure S1). The density of geraniol is 875 g/dm^3^, ${1/v}_{4}$ = 5.68 M, and the solubility limit in water is 4.65 mM. The (–)-Menthol was a product of TCI, Tokio, Japan (>99%, Cat. №. M0545) with $M_{w}$ = 156.27 g/mol; *ρ* = 890 g/dm^3^; ${1/v}_{4}$ = 5.70 M; and $C_{4}$ = 2.85 mM. The citronellol was a product of Sigma Aldrich, Merck KGaA, Burlington, MA, USA (> 95%, Cat. No. W230901): $M_{w}$ = 156.27 g/mol; *ρ* = 855 g/dm^3^; ${1/v}_{4}$ = 5.47 M; and $C_{4}$ = 1.55 mM. For more details, see Refs. [37,38].

The anionic surfactant sodium dodecyl sulfate (SDS) was a product of Sigma Aldrich, Merck KGaA, Burlington, MA, USA (>99.0% (GC) dust–free pellets) with $M_{w}$ = 288.38 g/mol; density at *ρ* = 1010 g/dm^3^; and ${1/v}_{1}$ = 3.50 M. All solutions contained 10 mM NaCl (Merck, Darmstadt, Germany) in order to minimize the effect of dodecanol, a hydrolysis product of SDS. The specific volume of the hydrated sodium ion is ${1/v}_{2}$ = 10.2 M.

In the case of fixed molar ratios between the SDS and a fragrance, the concentrations of the respective volatile amphiphiles in the stock aqueous solutions were chosen to be close to or equal to their solubility limit, $C_{4}$, in the presence of 10 mM NaCl. The stock solutions were stirred at room temperature and kept in a closed vessel at 25 °C in a thermostat for 24 h to ensure equilibration. Subsequently, these solutions (if lenses are not observed) were diluted with 10 mM NaCl to the desired concentrations. The diluted solutions were further thermostated at 25 °C for approximately 24 h prior to surface tension measurements. In the second case, stock aqueous solutions containing 0.2 mM SDS, 10 mM NaCl, and the volatile amphiphilic component were prepared and equilibrated under identical conditions. After equilibration, the stock solution was diluted with an aqueous solution containing 0.2 mM SDS and 10 mM NaCl, and thermostated at 25 °C, and the respective surface tension measurements were performed.

**Figure 10:**
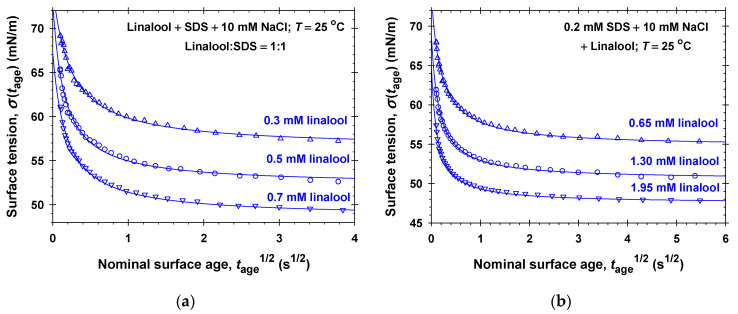
Dynamic surface tension vs. nominal surface age measured using MBPM for different linalool concentrations: (**a**) equimolar linalool and SDS concentrations in the presence of 10 mM NaCl; (**b**) 0.2 mM SDS + 10 mM NaCl with varying linalool concentrations. The solid lines show the best fits obtained using Equation (10), from which the equilibrium surface tensions, $\sigma_{eq}$, are calculated.

The dynamic surface tension of the surfactant and fragrance solutions was measured using the maximum bubble pressure method (MBPM) on the automated bubble pressure tensiometer BP 100 (Krüss GmbH, Hamburg, Germany). To obtain the equilibrium surface tension, $\sigma_{eq}$, we used the long-time asymptotic expansion equation [67]:

(10) $$\sigma (t_{\mathrm{age}})=\frac{b+\sigma_{\mathrm{eq}}t_{\mathrm{age}}^{1/2}}{a+t_{\mathrm{age}}^{1/2}},$$

where $\sigma (t_{age})$ is the dynamic surface tension measured at the nominal surface age, $t_{age}$, and *a* and *b* are constants. Equation (10) was derived for diffusion-controlled adsorption processes [67]. Figure 10 shows typical experimental data for $\sigma (t_{age})$ in the case of SDS + linalool aqueous solutions; the solid lines represent the best fits using Equation (10). The dependencies of the dynamic surface tension on the nominal surface age for mixtures of SDS and other fragrances are summarized in Figures S2–S4. In all cases, the values of the regression coefficients were greater than 0.9995 and the precision of the calculated equilibrium surface tension, $\sigma_{eq}$, was 0.1 mN/m. The good descriptions of the surface tension relaxations suggest that the mechanism of adsorption corresponds to diffusion control.

The fragrance molecules from the studied mixed solutions evaporate to the bubble phase due to the process of MBPM measurements. The experimental radius of all bubbles, $r_{b}$, is less than 0.2 mm and the diffusion coefficient of fragrance molecules in air is $D_{v}=6\times 10^{-6}$ m^2^/s [37,38]. The characteristic diffusion time of the fragrance molecules in bubbles becomes ${r_{b}^{2}/D}_{v}=6.7\;ms$. Thus, for experimental times longer than 0.1 s, the air in the bubbles is saturated with the fragrance molecules corresponding to the respective vapor pressure. As a result, the measured equilibrium surface tension corresponds to the interface between the mixed SDS + fragrance solution and the fragrance vapor phase, which is in equilibrium with the respective aqueous solution.

## 5. Conclusions

The surface tension isotherms of mixed fragrance (benzyl acetate, linalool, geraniol, menthol, and citronellol) and anionic sodium dodecyl sulfate (SDS) aqueous solutions measured at different solute ratios are excellently described by the van der Waals model for a two-component adsorption layer, taking into account the counterion binding in the Stern layer. The obtained best fit values of the effective interfacial pair interaction energy between adsorbed molecules ($\beta_{11}$, $\beta_{14}$, and $\beta_{44}$, see Table 1) show that in the adsorption layers, the attractive interaction energies between the adsorbed volatile amphiphilic and anionic surfactant molecules ($\beta_{14}$) are larger than those between the individual components ($\beta_{11}$ and $\beta_{44}$). Because of the different adsorption energies of fragrance molecules and SDS, the saturation adsorption of anionic surfactant becomes higher than the saturation adsorption of benzyl acetate, linalool, and geraniol, and, respectively, it is lower for menthol and citronellol even at equimolar mixed aqueous solutions.

The proposed generalized van der Waals model for mixed adsorption layers with the determined physicochemical parameters completely predicts the parameters of the interfacial phase: the surfactant, bound counterion, and fragrance adsorptions; the occupancy of the Stern layer by adsorbed counterions; the surface electrostatic potential; etc. (Figure 2 and Figure 5). The predictive power of the model is illustrated using the available experimental data for the linalool and SDS adsorptions in the presence of 100 mM NaCl and in the absence of added salt measured by neutron reflectivity [41] without adjustable parameters (Figure 8).

The processing of all experimental data for the critical aggregation concentration vs. mole fraction between fragrances and SDS (Figure 6) using the Rubingh theory gives information on the bulk interaction parameter, *β*, between volatile amphiphilic and anionic surfactant molecules (Table 1). We observed that: (i) the strongest antagonistic mixing (*β* > 0) takes place for citronellol + SDS, followed by benzyl acetate + SDS mixed aqueous solutions; (ii) the mixing of menthol and SDS is ideal; and (iii) the mixing of isomers linalool and geraniol and SDS is synergistic (*β* < 0).

The following general conclusions can be drawn from the detailed examination of the phase separation model for adsorption layers (PSMAL) previously applied in the literature to model mixed adsorption layers in the case of SDS + linalool [44] and SDS + geraniol [46] (see Figure 9). First, the binary mixed adsorption layers in the PSMAL are composed of fragrance molecules (component 4) and all other adsorbed ionic species are considered as component 1, which contains the contributions from the adsorption layer and the contiguous electrostatic double layer. Second, the PSMAL predicts the synergistic interactions between the anionic surfactant and fragrance molecules and the composition of the interfacial phase relatively well, but not the magnitudes of the adsorptions themselves.

The reported equilibrium interfacial and bulk properties of the mixed fragrance + SDS solutions are the starting point: (i) to construct the complex bulk phase diagrams of concentrated solutions [68,69,70,71] and (ii) to process the experimental data for the adsorption from fragrance vapor to SDS solution drop (desorption from saturated fragrance + SDS solution drop to the ambient atmosphere) in order to obtain the effect of surfactant on the fragrance condensation (evaporation) rate. The reported properties of the volatile amphiphiles and SDS mixtures could be of interest for increasing the range of their applicability in practice.

## Figures and Tables

**Table 1:** Interfacial and bulk physicochemical parameters for individual fragrances and for fragrance + SDS mixtures.

	Benzyl Acetate	Menthol	Linalool	Geraniol	Citronellol
$\alpha_{44}$ (Å^2^)	35.6	34.6	30.5	29.5	30.2
$E_{4}$ (*kT*)	6.64	9.51	9.05	9.07	9.80
$\beta_{44}$$\;(\alpha_{44}kT$)	2.05	1.57	0.965	1.92	2.52
*α*_14_ (Å^2^)	32.7	32.3	30.2	29.7	30.1
$\beta_{14}$$\;(\alpha_{14}kT$)	3.40 ± 0.03	3.00 ± 0.02	2.76 ± 0.02	3.60 ± 0.03	2.90 ± 0.03
*β*	1.17 ± 0.02	0.0	−0.268 ± 0.005	−0.348 ± 0.003	1.60 ± 0.05

## Data Availability

Data are contained within the article or the Supplementary Material. The original contributions presented in this study are included in the article/Supplementary Material. Further inquiries can be directed to the corresponding author.

## Supplementary Materials

The following supporting information can be downloaded at: https://www.mdpi.com/article/10.3390/molecules31081256/s1, Figure S1: Chemical structures of the used materials; Figure S2: Dynamic surface tension vs. nominal surface age measured using MBPM for different geraniol concentrations; Figure S3: Dynamic surface tension vs. nominal surface age measured using MBPM for different menthol concentrations; Figure S4: Dynamic surface tension vs. nominal surface age measured using MBPM for different benzyl acetate concentrations; Figure S5: (a) Surface tension isotherms of 0.2 mM SDS + 10 mM NaCl and fragrance (geraniol and menthol) mixed solutions: symbols—experimental data; solid lines show the calculated best fit results. Calculated (b) surfactant and fragrance adsorptions, (c) occupancy of the Stern layer, and (d) surface electrostatic potentials vs. fragrance concentrations; Figure S6: Calculated dependencies of (a) the surfactant adsorption, (b) the fragrance adsorption, (c) the occupancy of the Stern layer, and (d) the surface electrostatic potentials as functions on the total concentration for fragrance + SDS solutions in the presence of 10 mM NaCl at fixed molar ratios between the fragrance (benzyl acetate, linalool, and citronellol) and SDS; Figure S7: Surface tension isotherms of menthol + SDS solutions in the presence of 10 mM NaCl at fixed molar ratios between menthol and SDS. The symbols are experimental data and the solid lines represent the best fits by means of our model; Figure S8: Calculated dependencies of (a) the surfactant adsorption, (b) the fragrance adsorption, (c) the occupancy of the Stern layer, and (d) the surface electrostatic potentials on the total concentration for fragrance + SDS solutions in the presence of 10 mM NaCl at fixed molar ratios between the fragrance (menthol and geraniol) and SDS; Figure S9: (a) Mole fractions, $y_{1}$ and $y_{4}$, vs. SDS bulk mole fractions for linalool + SDS and citronellol + SDS mixed solutions. (b) Activity coefficients, $\gamma_{1}$ and $\gamma_{4}$, vs. SDS bulk mole fraction for geraniol + SDS and linalool + SDS mixed solutions; Figure S10: Fragrance mole fraction in the adsorption layers, $y_{4\sigma }$, vs. the SDS bulk mole fraction, $x_{1}$, for linalool + SDS: (a) *σ* = 55 mN/m; (b) *σ* = 60 mN/m; (c) *σ* = 65 mN/m; Figure S11: Fragrance mole fraction in the adsorption layers, $y_{4\sigma }$, vs. the SDS bulk mole fraction, $x_{1}$, for geraniol + SDS: (a) *σ* = 55 mN/m; (b) *σ* = 60 mN/m; (c) *σ* = 65 mN/m.
