# Supplementary material for: Interfacial and Bulk Properties of Volatile Amphiphiles and Sodium Dodecyl Sulfate Mixtures
Source: Molecules. 2026 Apr 10;31(8):1256. doi: 10.3390/molecules31081256 (PMC13118941; doi:10.3390/molecules31081256)
Supplement: Supplementary file 1 [file molecules-31-01256-s001.zip › molecules-4219389-supplementary.pdf]

Interfacial and Bulk Properties of Volatile Amphiphiles and Sodium Dodecyl Sulfate Mixtures

Ralitsa Uzunova [1,2], Rumyana Stanimirova [1,2] and Krassimir Danov [1,2,*]

1 Department of Chemical & Pharmaceutical Engineering, Faculty of Chemistry & Pharmacy, Sofia University “St. Kliment Ohridski”, 1164 Sofia, Bulgaria; ru@lcpe.uni-sofia.bg (R.U.); rs@lcpe.uni-sofia.bg (R.S.)
2 CoC “Smart Mechatronics, Eco- and Energy Saving Systems and Technologies”, 1164 Sofia, Bulgaria
* Correspondence: kd@lcpe.uni-sofia.bg

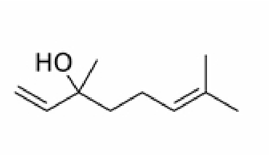

(**a**)

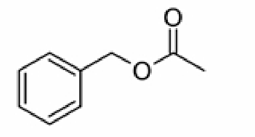

(**b**)

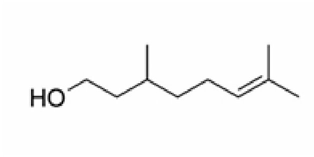

(**c**)

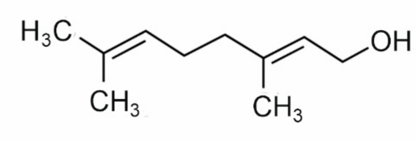

(**d**)

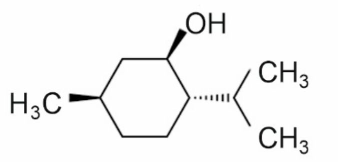

(**e**)

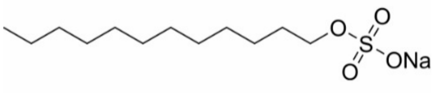

(**f**)

Figure S1. Chemical structures of the used materials: **(a)** linalool; **(b)** benzyl acetate; **(c)** citronellol; **(d)** geraniol; **(e)** (–)-menthol; **(f)** sodium dodecyl sulfate. (SDS).

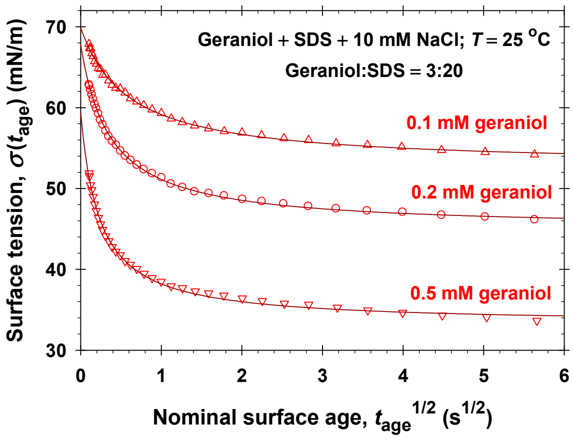

(**a**)

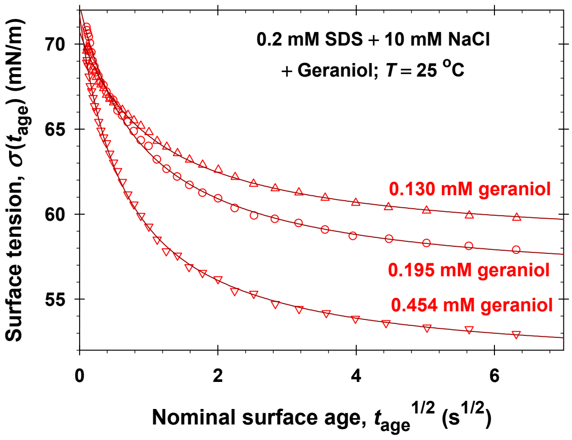

(**b**)

Figure S2. Dynamic surface tension vs nominal surface age measured using MBPM for different geraniol concentrations: (**a**) geraniol + SDS (3:20) in the presence of 10 mM NaCl; (**b**) 0.2 mM SDS + 10 mM NaCl and different concentrations of geraniol. The solid lines show the best fits obtained using Equation (1), from which the equilibrium surface tensions, σ_{eq}, are calculated.

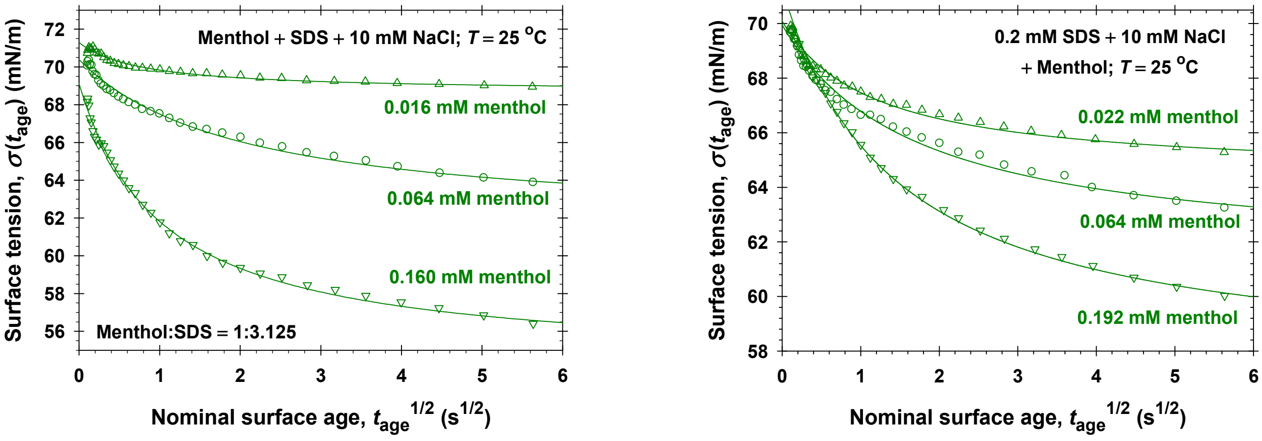

(**a**) (**b**)

Figure S3. Dynamic surface tension vs nominal surface age measured using MBPM for different menthol concentrations: (**a**) menthol + SDS (1:3.125) in the presence of 10 mM NaCl; (**b**) 0.2 mM SDS + 10 mM NaCl and different concentrations of menthol. The solid lines show the best fits obtained using Equation (1), from which the equilibrium surface tensions, σ_{eq}, are calculated.

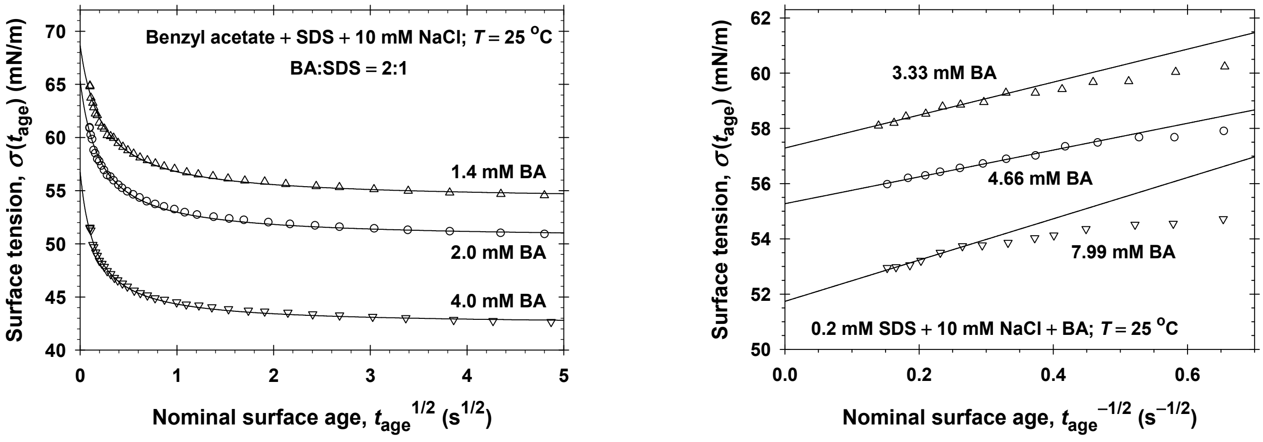

(**a**) (**b**)

Figure S4. Dynamic surface tension vs nominal surface age measured using MBPM for different benzyl acetate (BA) concentrations: (**a**) benzyl acetate + SDS (2:1) in the presence of 10 mM NaCl – the solid lines show the best fits obtained using Equation (1); (**b**) 0.2 mM SDS + 10 mM NaCl and different concentrations of benzyl acetate (BA) – the solid lines show the inverse square of time asymptotes.

For 1:1 symmetric electrolytes, the ionic strength, I, is calculated from the following formula: $2I = c_1 + c_2 + c_3 = 2c_2$. In the literature, the following semi-empirical formula,

$$\log_{10} \gamma_{\pm} = 0.055I - \frac{0.5115\sqrt{I}}{1+1.316\sqrt{I}}, \quad \text{(S1)}$$

is widely used to process experimental data. In Equation (S1), the ionic strength, I, is given in M.

The definition of the Bjerrum length, λ, is:

$$\lambda \equiv \frac{e^2}{4\pi\varepsilon_0\varepsilon kT}. \quad \text{(S2)}$$

Here: k is the Boltzmann constant; T is the absolute temperature; e is the electronic charge; ε_0 is the dielectric permittivity of vacuum; ε is the relative dielectric permittivity of the solution.

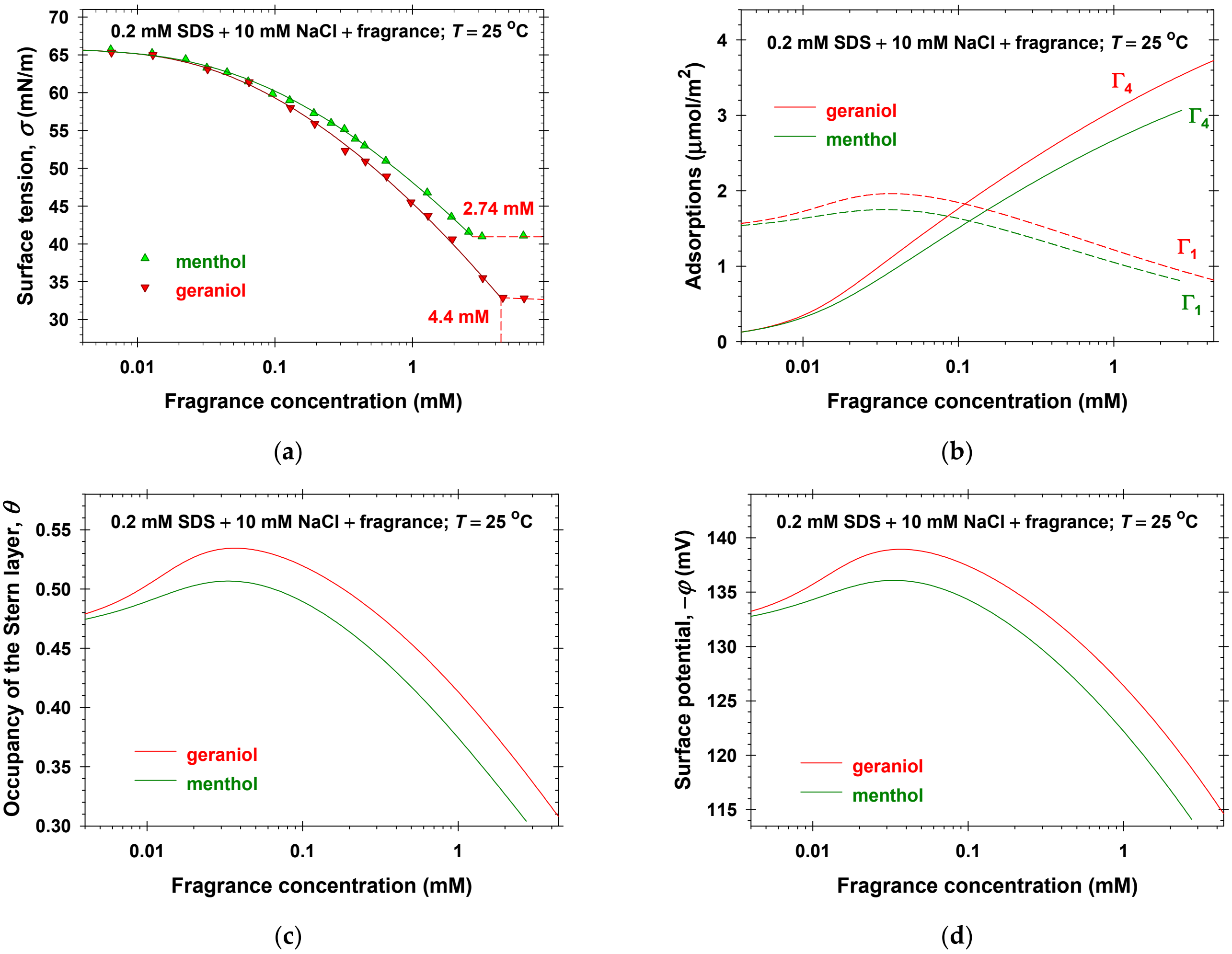

Figure S5. (**a**) Surface tension isotherms of 0.2 mM SDS + 10 mM NaCl and fragrance (geraniol and menthol) mixed solutions: symbols – experimental data; solid lines show the calculated best fit results. Calculated (**b**) surfactant and fragrance adsorptions, (**c**) occupancy of the Stern layer, and (**d**) surface electrostatic potentials vs fragrance concentrations.

The experimental data for the surface tension vs the fragrance concentration in the case of mixed 0.2 mM SDS + 10 mM NaCl + fragrance aqueous solutions at different menthol and geraniol concentrations are shown in Figure S5a. The solid lines therein correspond to the best fits using our model with the physicochemical parameters listed in Table 1. The respective calculated data for the adsorptions (Figure S5b), the occupancy of the Stern layer (Figure S5c), and the surface electrostatic potential (Figure S5d) show the differences of the properties of the adsorption layers in the case of menthol and geraniol. The respective surface tension isotherms of menthol + SDS aqueous solutions in the presence of 10 mM NaCl at fixed molar ratios between menthol and SDS are summarized in Figure S7.

Figure S6 illustrates the effects of the fragrance type (benzyl acetate, linalool, and citronellol) on the properties of mixed adsorption layers as functions of the total species concentrations for fragrance + SDS aqueous solutions in the presence of 10 mM NaCl at fixed molar ratios between the fragrance and the anionic surfactant. The analogous

properties of the mixed adsorption layer in the case of menthol and geraniol are summarized in Figure S8.

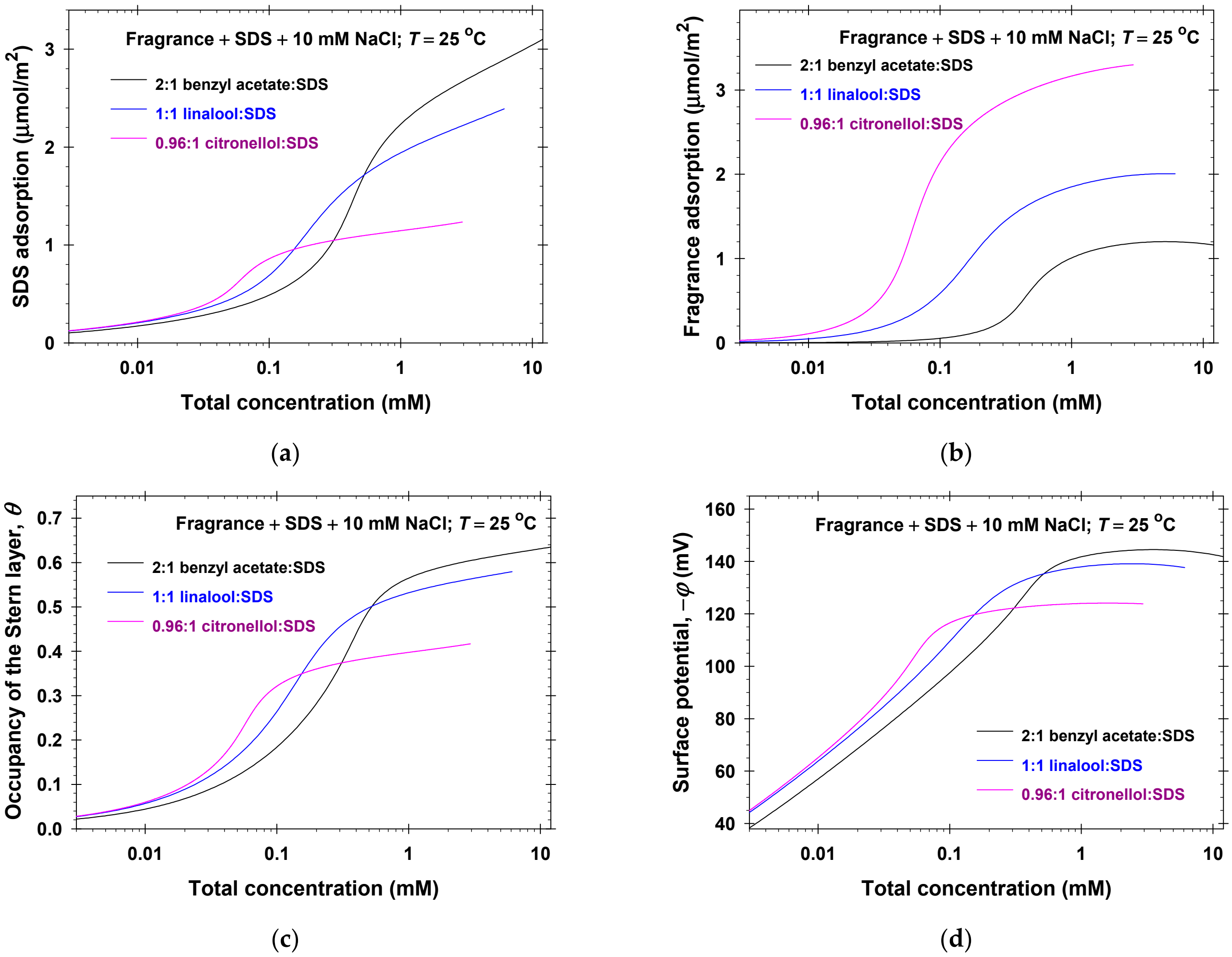

Figure S6. Calculated dependencies of (**a**) the surfactant adsorption, (**b**) the fragrance adsorption, (**c**) the occupancy of the Stern layer, and (**d**) the surface electrostatic potentials as functions on the total concentration for fragrance + SDS solutions in the presence of 10 mM NaCl at fixed molar ratios between the fragrance (benzyl acetate, linalool, and citronellol) and SDS.

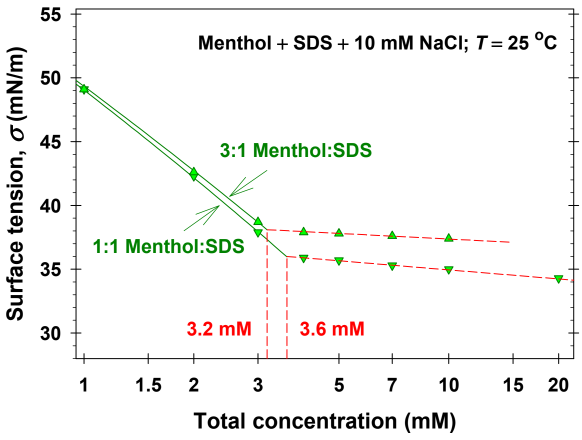

Figure S7. Surface tension isotherms of menthol + SDS solutions in the presence of 10 mM NaCl at fixed molar ratios between menthol and SDS. The symbols are experimental data and the solid lines represent the best fits by means of our model.

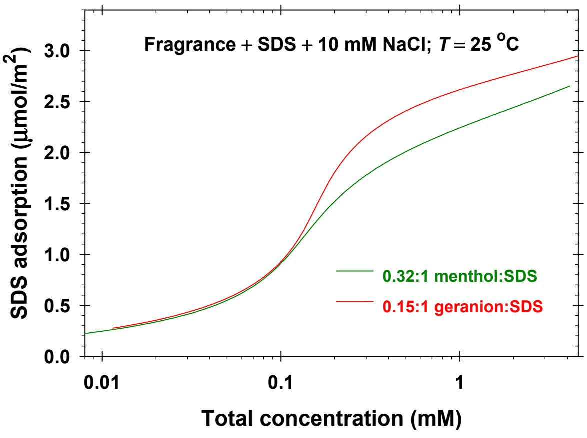

(**a**)

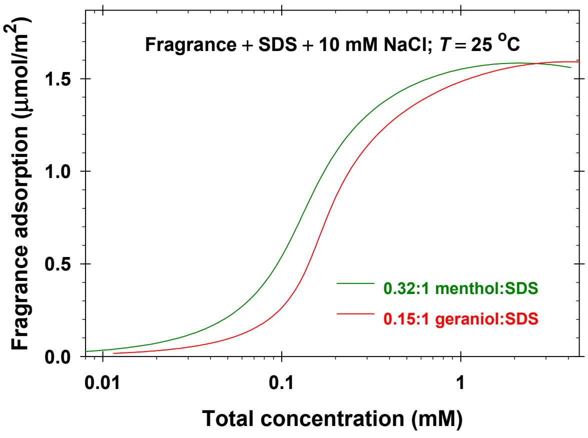

(**b**)

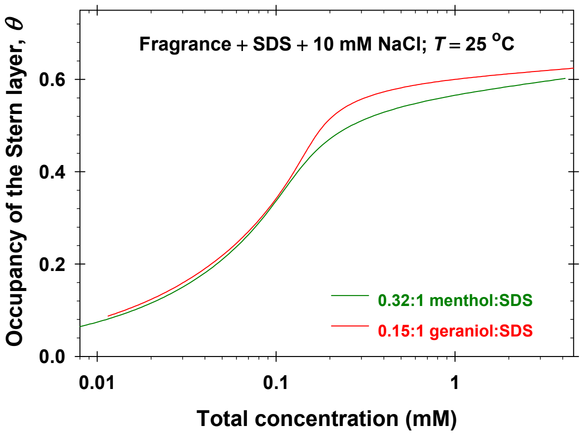

(**c**)

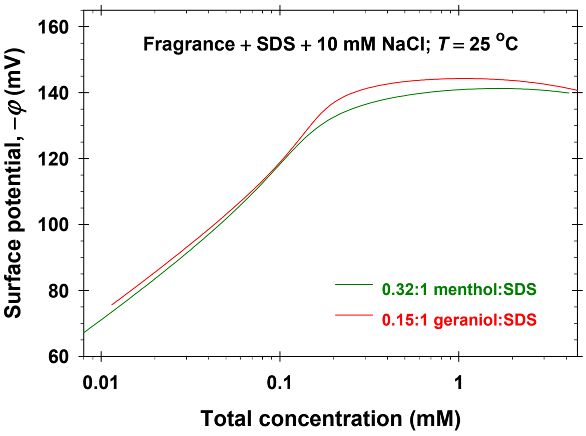

(**d**)

Figure S8. Calculated dependencies of (**a**) the surfactant adsorption, (**b**) the fragrance adsorption, (**c**) the occupancy of the Stern layer, and (**d**) the surface electrostatic potentials on the total concentration for fragrance + SDS + 10 mM NaCl solutions at fixed molar ratios between the fragrance (menthol and geraniol) and SDS.

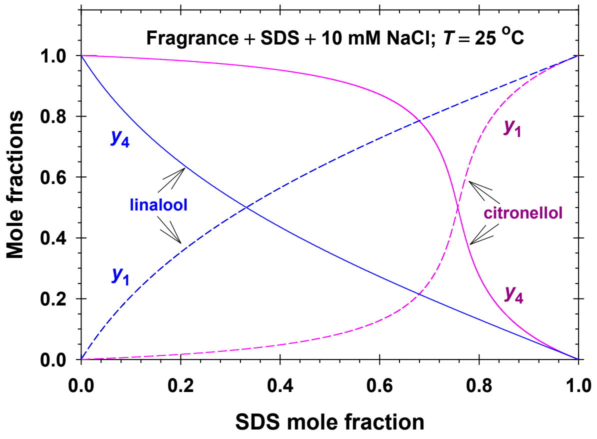

(**a**)

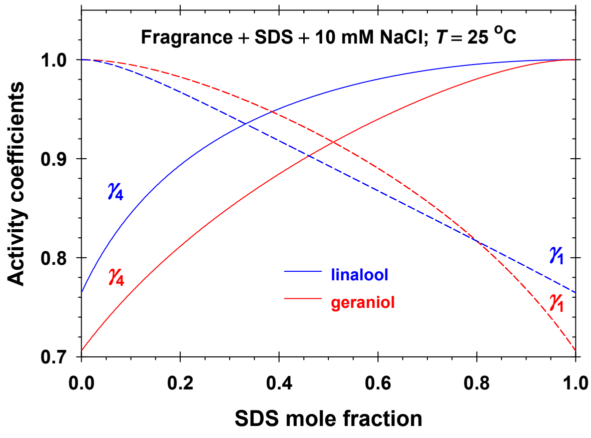

(**b**)

Figure S9. (**a**) Mole fractions, y_1 and y_4, vs SDS bulk mole fractions for linalool + SDS and citronellol + SDS mixed solutions. (**b**) Activity coefficients, γ_1 and γ_4, vs SDS bulk mole fraction for geraniol + SDS and linalool + SDS mixed solutions.

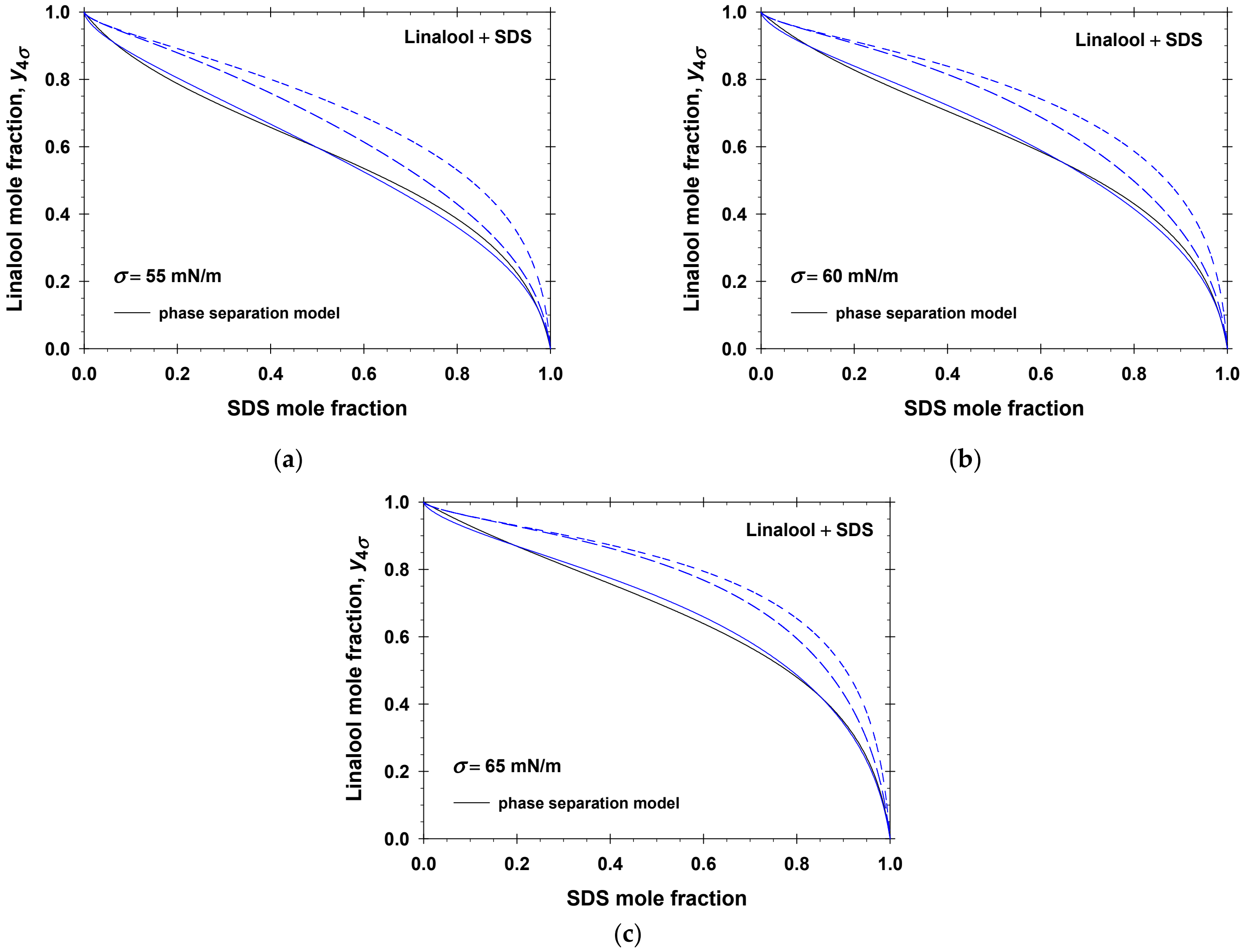

Figure S10. Fragrance mole fraction in the adsorption layers, $y_{4\sigma}$, vs the SDS bulk mole fraction, x_1, for linalool + SDS: (**a**) σ = 55 mN/m; (**b**) σ = 60 mN/m; (**c**) σ = 65 mN/m.

In the Rosen theory for binary adsorption layers, the properties of the fragrance molecules in the interfacial phase are characterized by their mole fraction $y_{4\sigma}$ and activity coefficient $\gamma_{4\sigma}$. All other adsorbed species have mole fraction $y_{1\sigma}$ and activity coefficient $\gamma_{1\sigma}$, where $y_{1\sigma} + y_{4\sigma} = 1$. At a constant temperature and two-dimensional surface pressure (i.e., surface tension σ), the chemical equilibrium between the bulk monomers and the adsorbed molecules in the interfacial phase with respect to the exchange of molecules types "1" and "4" yields:

$$\ln\left(x_1 c_\sigma\right) = \ln c_{1\sigma} + \ln\left(\gamma_{1\sigma} y_{1\sigma}\right);\ \ln\left(x_4 c_\sigma\right) = \ln c_{4\sigma} + \ln\left(\gamma_{4\sigma} y_{4\sigma}\right). \qquad \text{(S3)}$$

Here: $c_{1\sigma}$ and $c_{4\sigma}$ are the concentrations of individual components corresponding to a defined two-dimensional pressure; c_σ is the total concentration of anionic surfactant and fragrance at different bulk mole fractions x_1 and x_4 corresponding to the same fixed value of σ. By analogy with the Rubingh theory, the activity coefficients are expressed in the form:

$$\gamma_{1\sigma} = \exp\left(\beta_\sigma y_{2\sigma}^2\right);\ \gamma_{2\sigma} = \exp\left(\beta_\sigma y_{1\sigma}^2\right). \qquad \text{(S4)}$$

In Equation (S4), the surface interaction parameter, β_σ, does not depend on the bulk composition but can be different for different values of the two-dimensional surface pressure, that is on the surface tension. If one accounts for the interaction energies of

components listed in Table 1, then the approximate value of β_σ can be simply calculated as follows:

$$\beta_\sigma \approx \frac{1}{2kT}\left(\frac{\beta_{11}}{\alpha_{11}} + \frac{\beta_{44}}{\alpha_{44}} - 2\frac{\beta_{14}}{\alpha_{14}}\right). \tag{S5}$$

Equation (S5) is not exact because it accounts only for the long-range interactions between molecules in the interfacial phase.

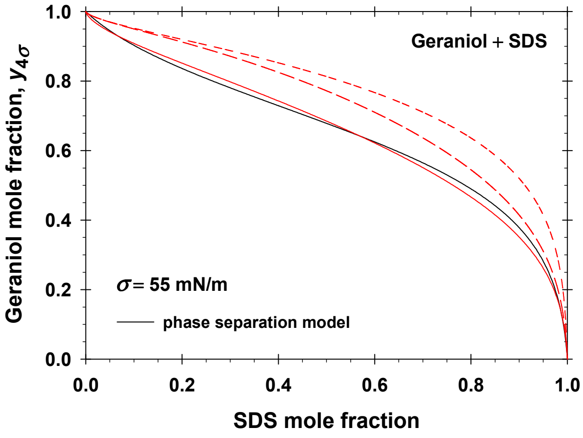

(**a**)

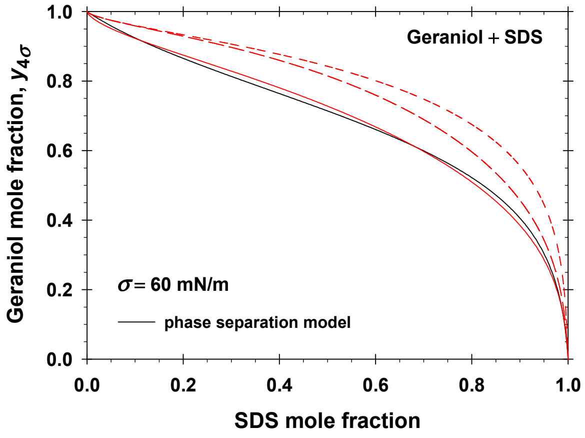

(**b**)

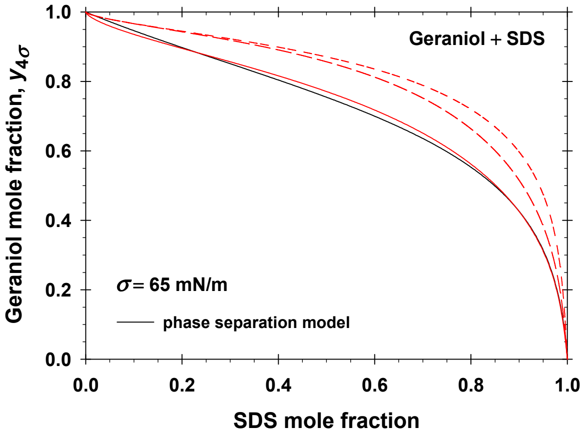

(**c**)

Figure S11. Fragrance mole fraction in the adsorption layers, $y_{4\sigma}$, vs the SDS bulk mole fraction, x_1, for geraniol + SDS: (**a**) σ= 55 mN/m; (**b**) σ= 60 mN/m; (**c**) σ= 65 mN/m.
